# Supplementary material for: Anti-Tumor and Anti-Inflammatory Activity In Vivo of Apodanthera congestiflora Cogn. (Cucurbitaceae)
Source: Pharmaceutics. 2021 May 18;13(5):743. doi: 10.3390/pharmaceutics13050743 (PMC8157552; doi:10.3390/pharmaceutics13050743)
Supplement: Supplementary file 1 [file pharmaceutics-13-00743-s001.zip › pharmaceutics-1207278 -supplementary final.pdf]

pharmaceutics

Supplementary Materials: Anti-Tumor and Anti-Inflammatory Activity In Vivo of *Apodanthera congestiflora* Cogn. (Cucurbitaceae)

Geovana F. G. Silvestre, Renally P. Lucena, Genil D. Oliveira, Helimarcos N. Pereira, Jhonatta A. B. Dias, Ivone A. Souza and Harley S. Alves

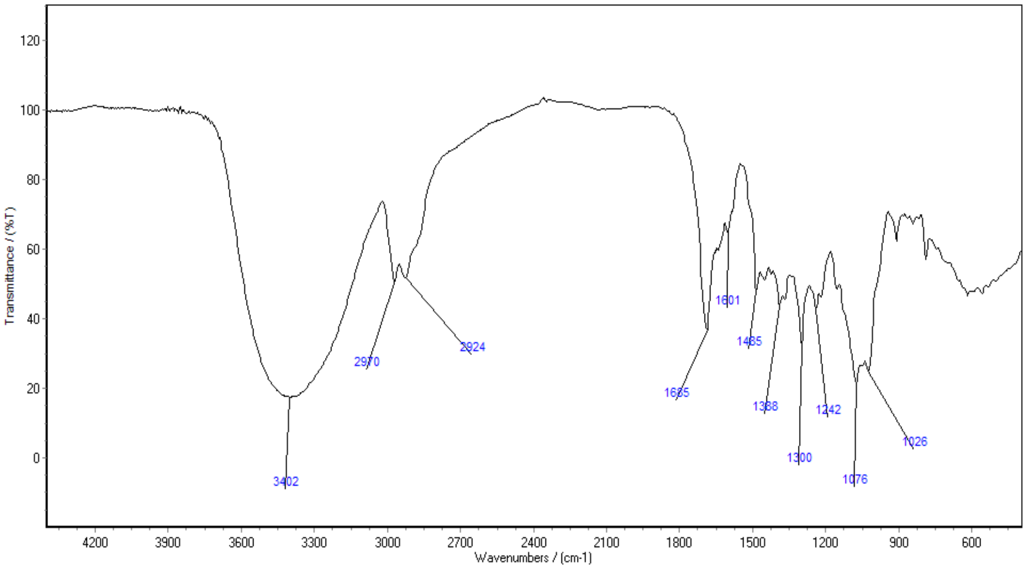

Figure S1. Infrared spectrum of the mixture of Ac-1, Ac-2 and Ac-3.

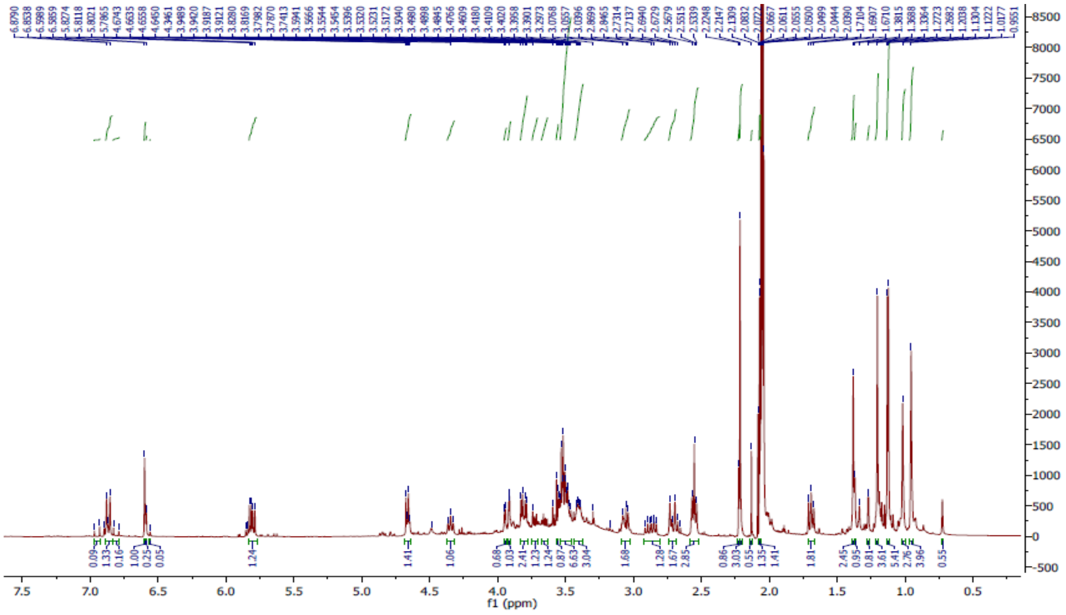

Figure S2. ^{1}H NMR spectrum (δ, acetone-d6, 400 MHz) of Ac-1, Ac-2 and Ac-3.

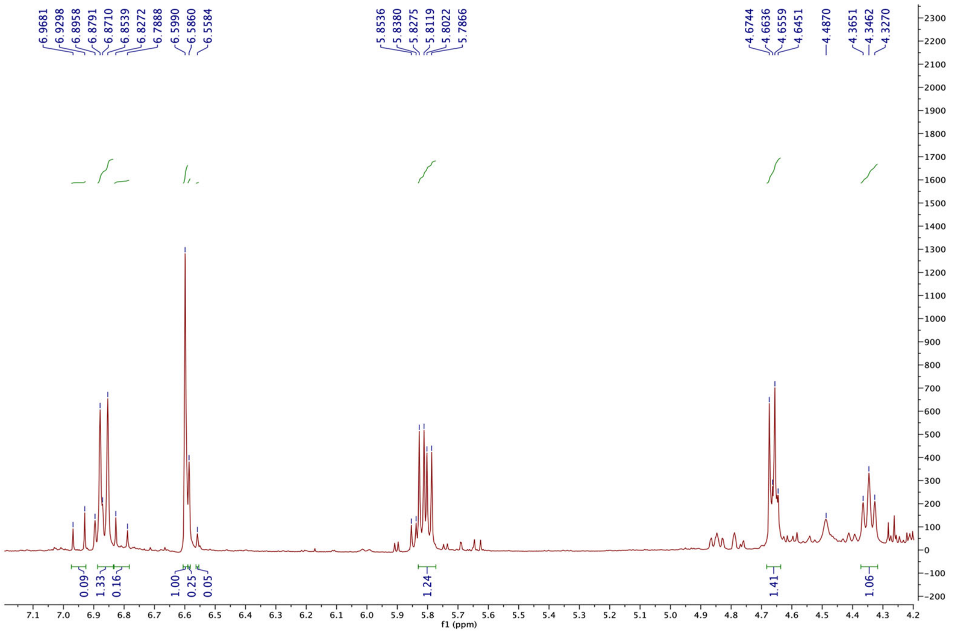

Figure S3. ¹H NMR spectrum expansion (δ, acetone-d6, 400 MHz) of Ac-1, Ac-2 and Ac-3**.**

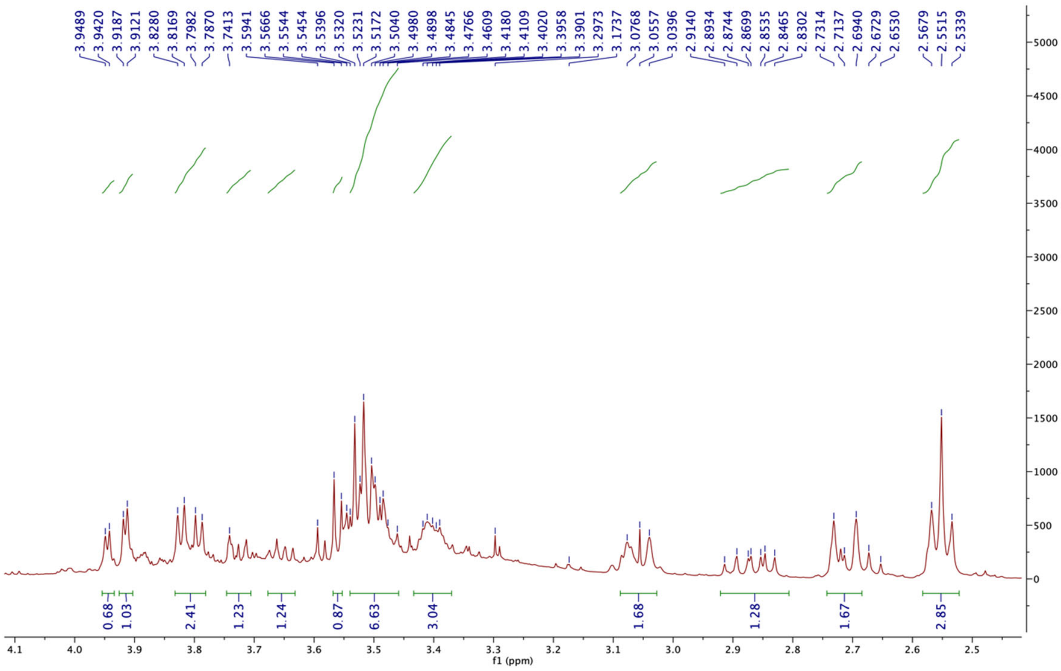

Figure S4. ¹H NMR spectrum expansion (δ, acetone-d6, 400 MHz) of Ac-1, Ac-2 and Ac-3.

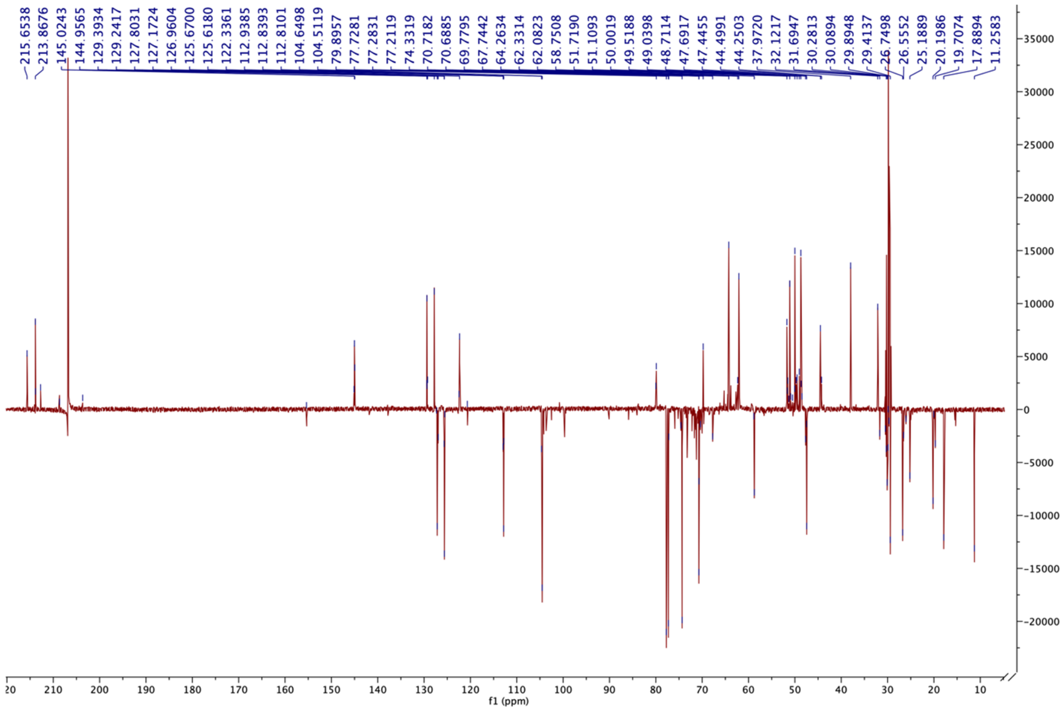

Figure S5. ^{13}C NMR spectrum (δ, acetone-d6, 100 MHz) of Ac-1, Ac-2 and Ac-3.

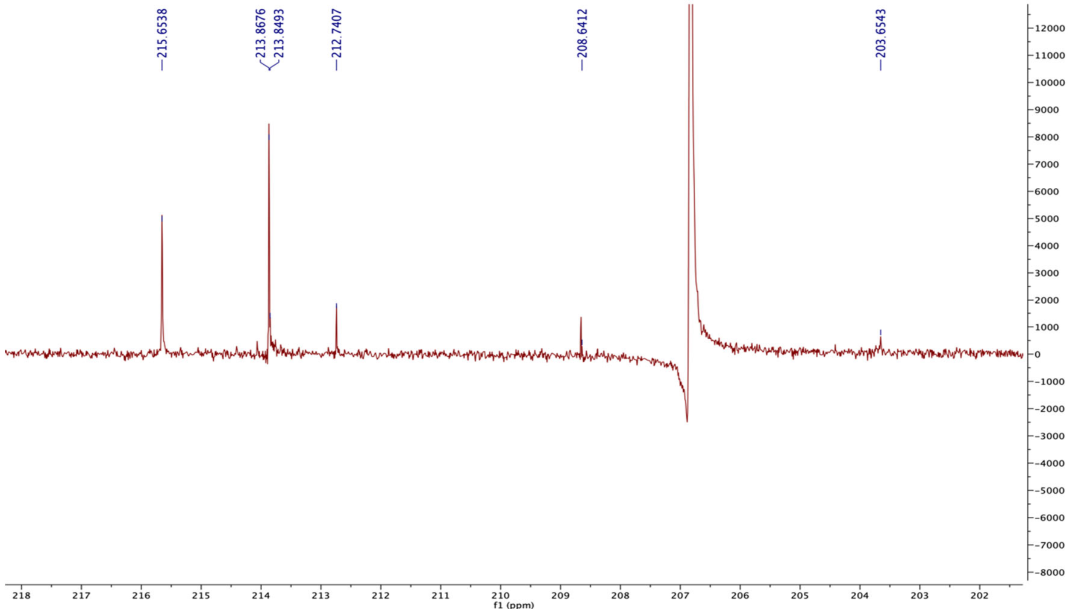

Figure S6. ^{13}C NMR spectrum expansion (δ, acetone-d6, 100 MHz) of Ac-1, Ac-2 and Ac-3.

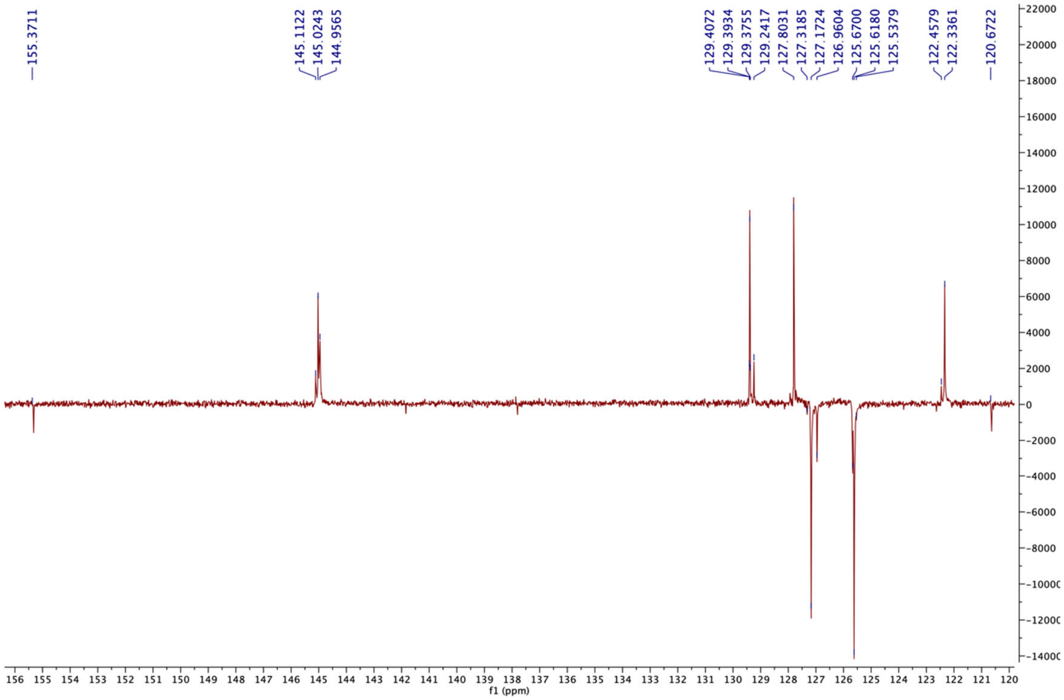

Figure S7. ^{13}C NMR spectrum expansion (δ, acetone-d6, 100 MHz) of Ac-1, Ac-2 and Ac-3.

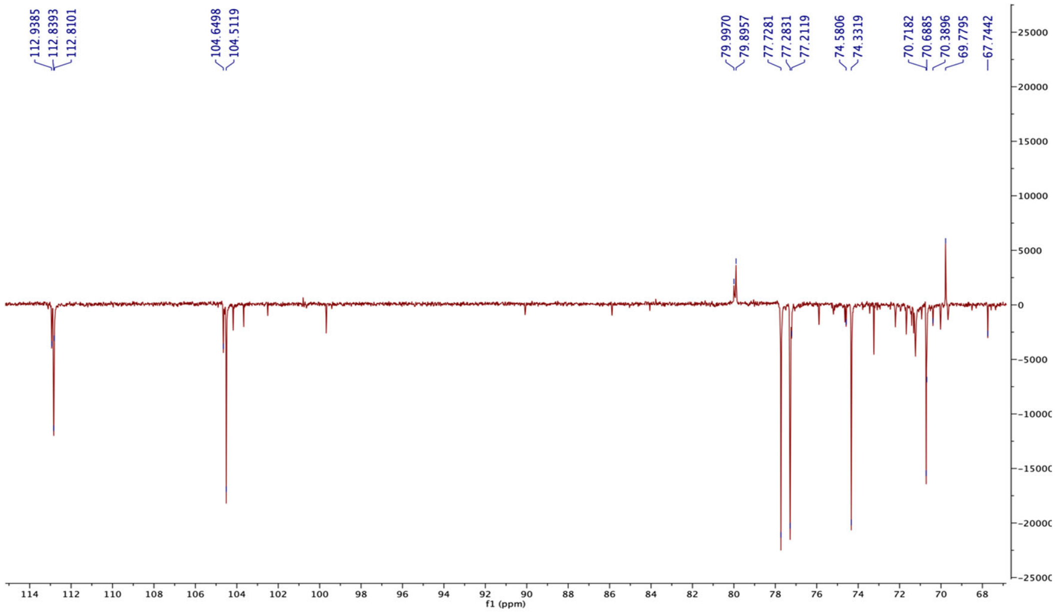

Figure S8. ^{13}C NMR spectrum expansion (δ, acetone-d6, 100 MHz) of Ac-1, Ac-2 and Ac-3.

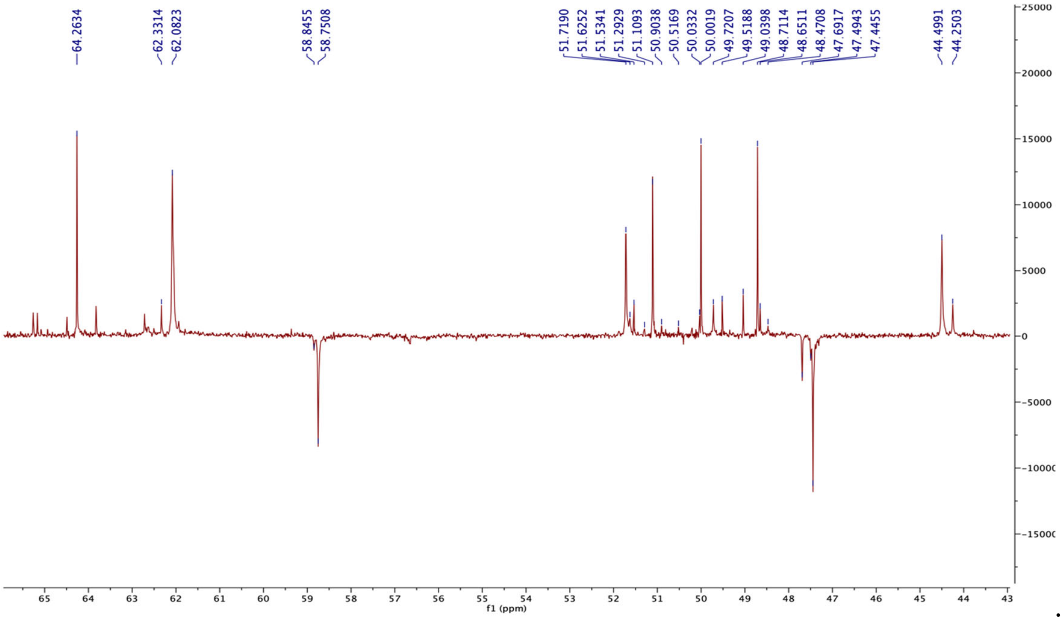

Figure S9. ^{13}C NMR spectrum expansion (δ, acetone-d6, 100 MHz) of Ac-1, Ac-2 and Ac-3.

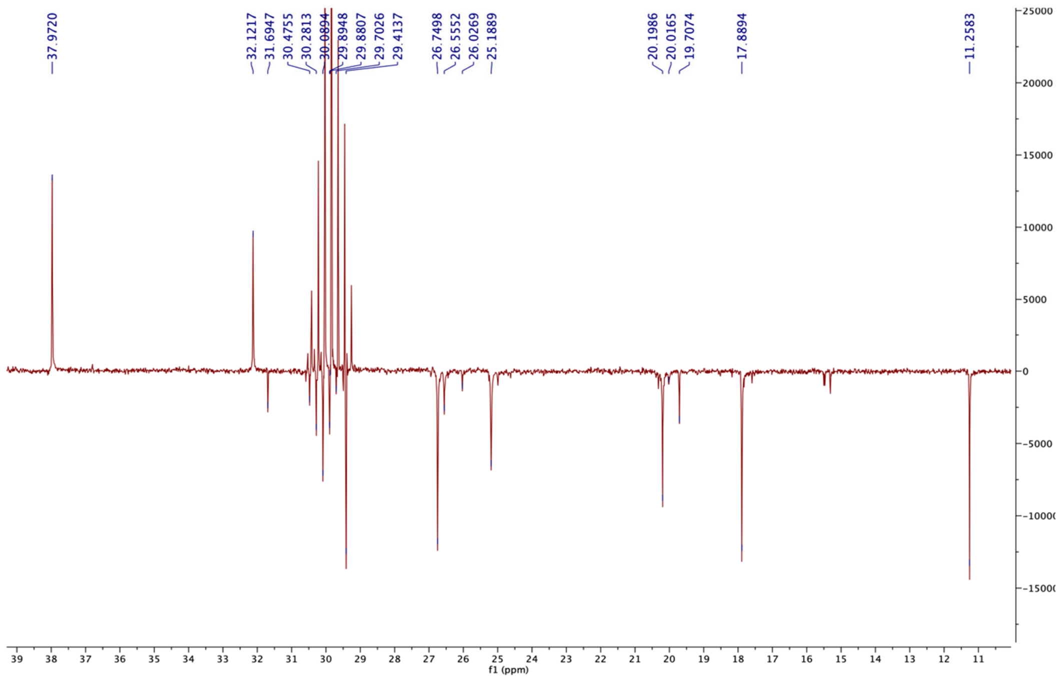

Figure S10. ^{13}C NMR spectrum expansion (δ, acetone-d6, 100 MHz) of Ac-1, Ac-2 and Ac-3.

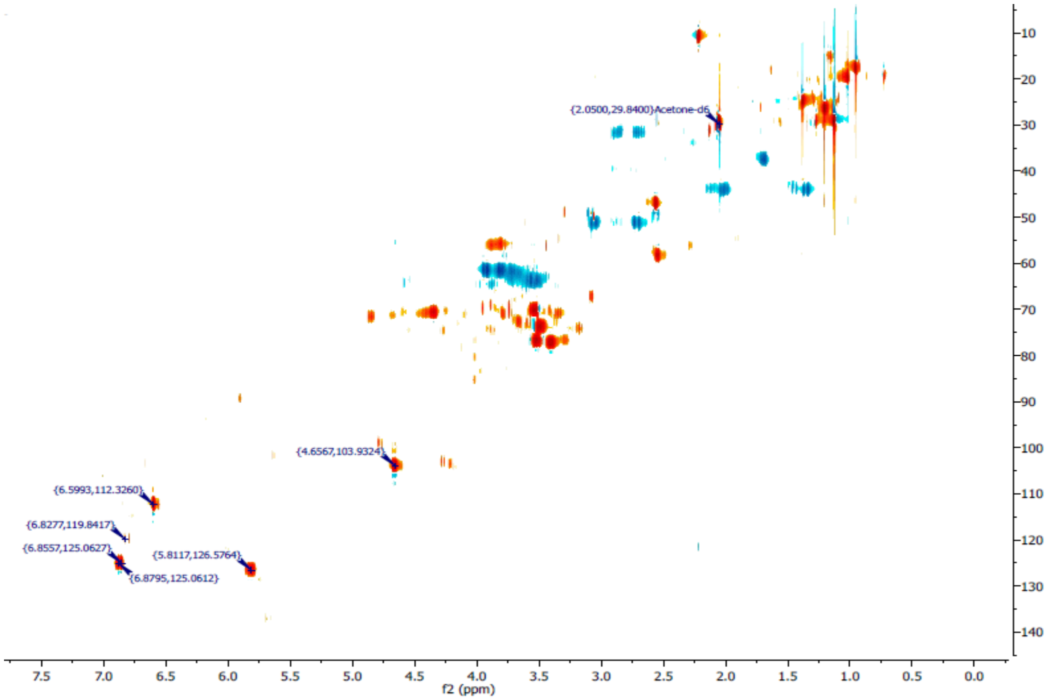

Figure S11. HSQC contour map – ^{1}H x ^{13}C of Ac-1, Ac-2 and Ac-3.

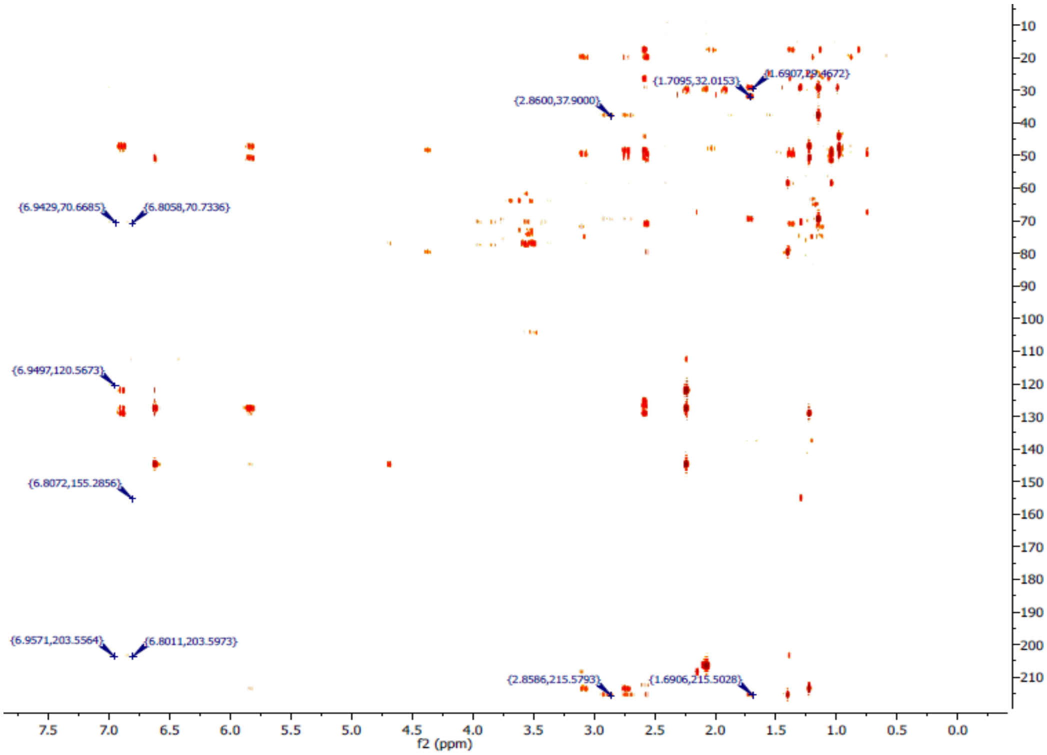

Figure S12. HMBC contour map – ^{1}H x ^{13}C of Ac-1, Ac-2 and Ac-3.

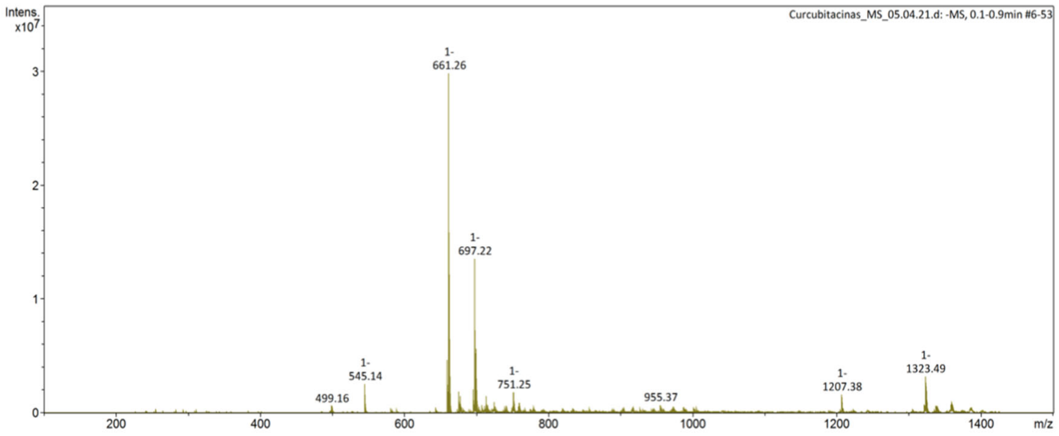

Figure S13. Mass spectrum of Ac-1.

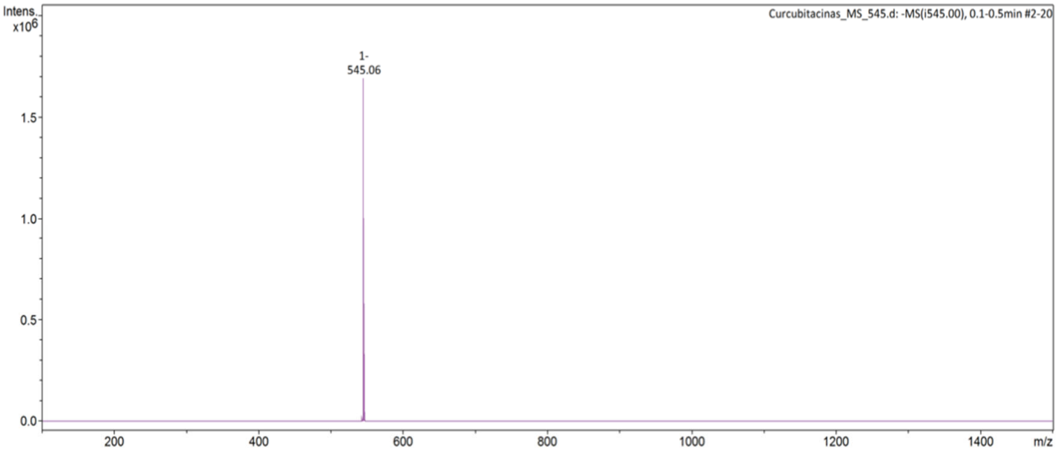

Figure S14. Mass spectrum of Ac-2.

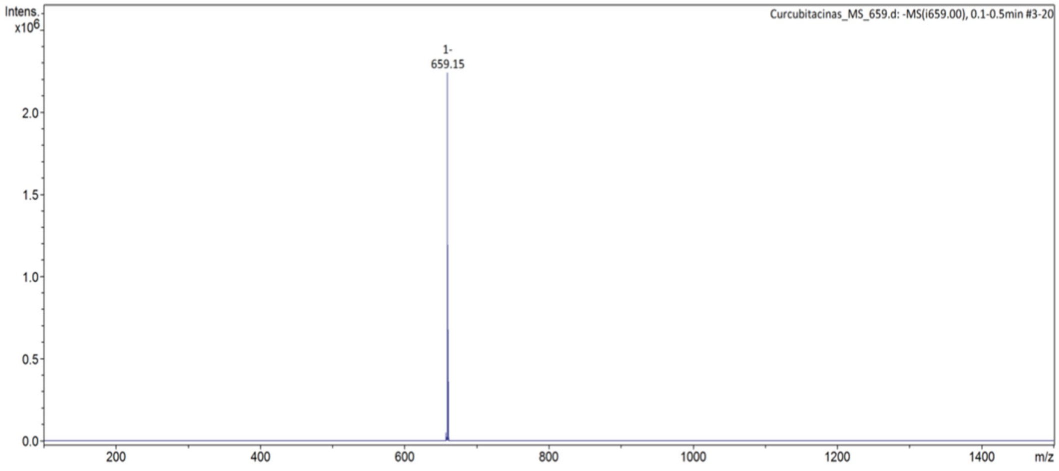

Figure S15. Mass spectra of Ac-3.

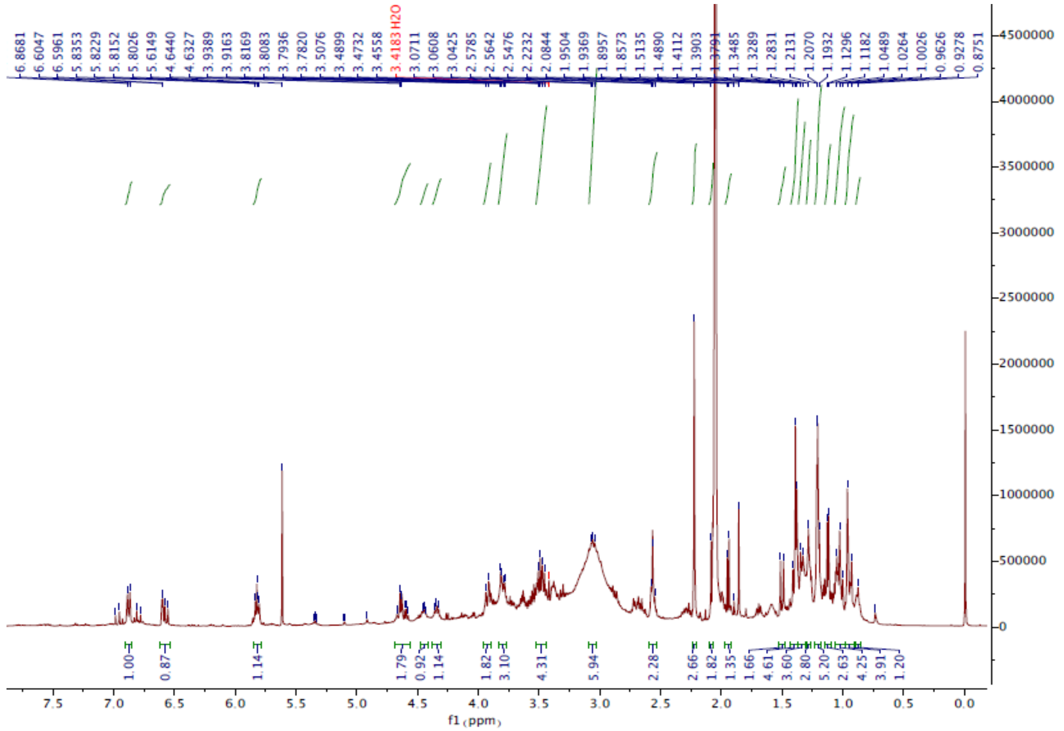

Figure S16. ^{1}H NMR spectrum (δ, acetone-d_6, 500 MHz) of dichloromethane phase.

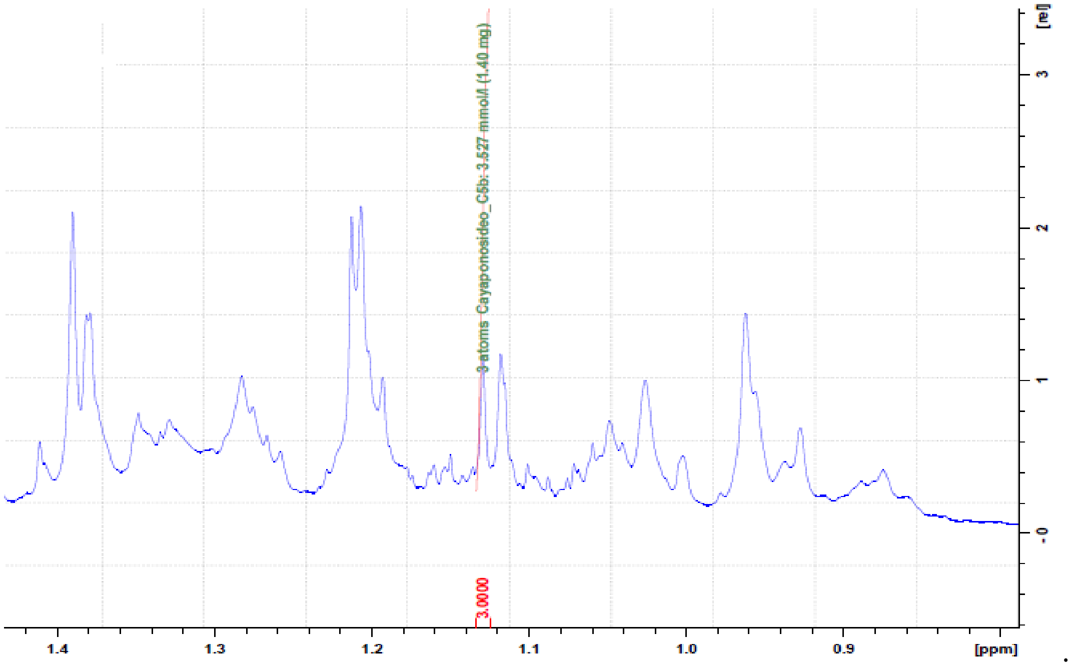

Figure S17. ^{1}H NMR spectrum expansion (δ, acetone-d_6, 500 MHz) of dichloromethane phase showing the region of terminal methyl at δ_H 1.13 analyzed by the TopicSpin Eretic—analysis 1.

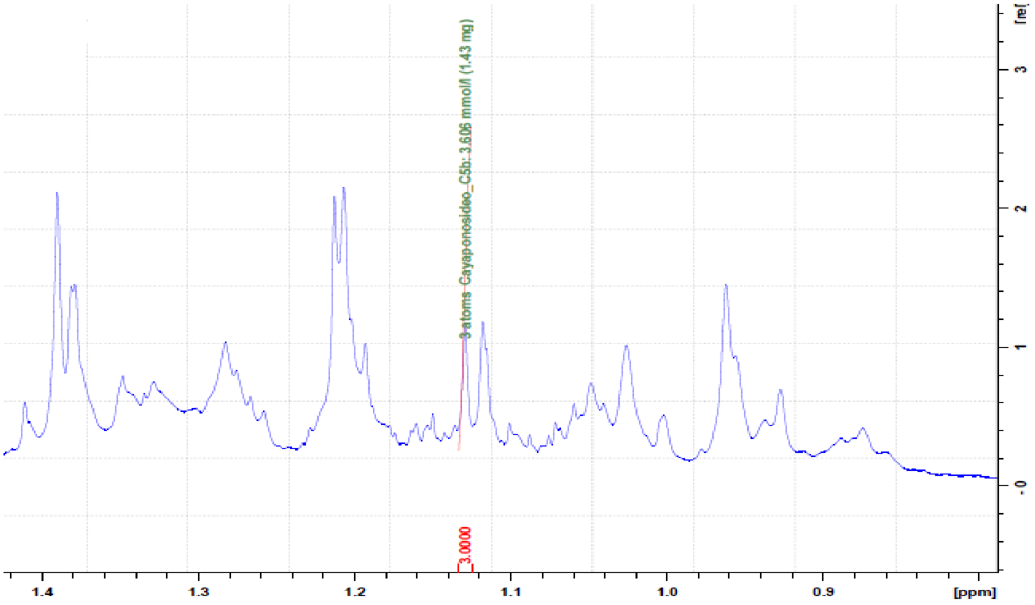

Figure S18. ^{1}H NMR spectrum expansion (δ, acetone-d_6, 500 MHz) of dichloromethane phase showing the region of terminal methyl at δ_H 1.13 analyzed by the TopicSpin Eretic—analysis 2.

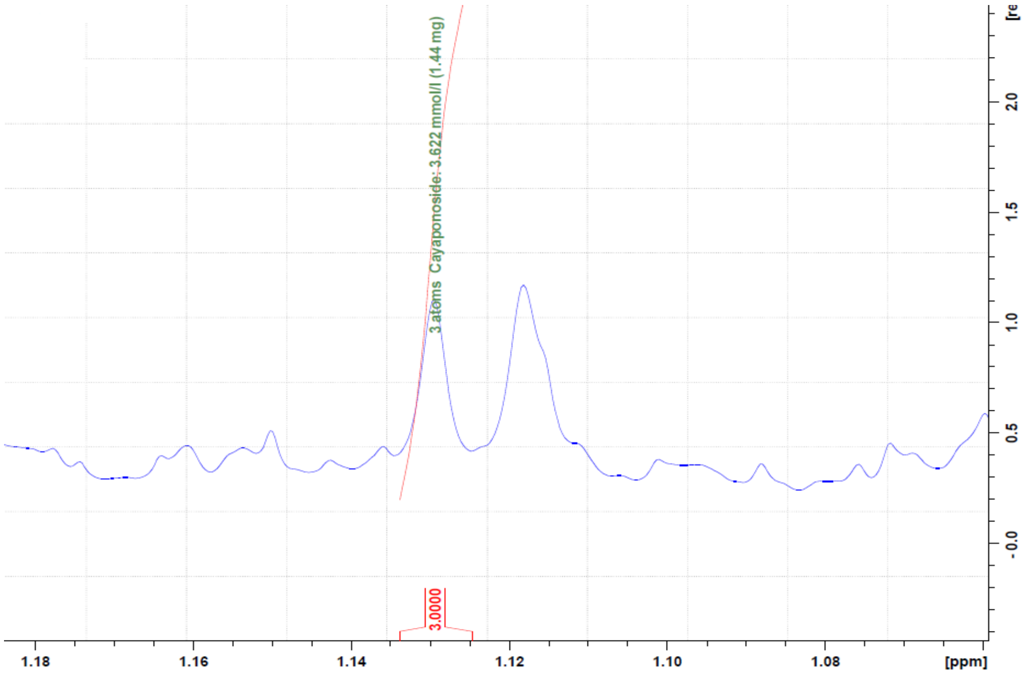

Figure S19. ^{1}H NMR spectrum expansion (δ, acetone-d_6, 500 MHz) of dichloromethane phase showing the region of terminal methyl at δ_H 1.13 analyzed by the TopicSpin Eretic—analysis 3.
